# A Case Report of the Clinico-radiologic Challenges of Assessing Treatment Response after Stereotactic Radiation of Oligometastases Preceded by Immunotherapy: Pseudoprogression, Mixed Response Patterns, and Opportunities for Precision Radiation

**DOI:** 10.7759/cureus.4264

**Published:** 2019-03-18

**Authors:** Arif S Rashid, Sriram Venigalla, Michael Dzeda, Gregory A Masters

**Affiliations:** 1 Radiation Oncology, Hahnemann University Hospital, Philadelphia, USA; 2 Radiation Oncology, University of Pennsylvania Perelman School of Medicine, Philadelphia, USA; 3 Radiation Oncology, Helen F. Graham Cancer Center – Christiana Care Health System, Newark, USA; 4 Oncology, Helen F. Graham Cancer Center – Christiana Care Health System, Newark, USA

**Keywords:** radiation oncology, immunotherapy, oligometastasis, stereotactic radiation, pseudoprogression, immune check point inhibitors, hyperprogression, abscopal effect

## Abstract

As immunotherapy continues to translate to the clinic and is combined with existing modalities, such as radiation therapy, novel treatment response patterns have been observed which complicate conventional clinical assessment and management. Herein, we describe a case study of a patient with non-small cell lung cancer treated initially with definitive chemoradiation who subsequently developed oligorecurrent disease which was managed with nivolumab and then comprehensive salvage stereotactic radiation. Serial radiographic assessment had shown worsening at these limited sites of disease after initiating immunotherapy, improvement after radiation, and then heterogeneous response behavior across sites during longer-term follow-up. Given the dual effects ablative radiation may have in the context of global immune checkpoint inhibition, both cytotoxic and synergistic immune-related, assessment of treatment response to such treatment is complicated. Such assessment is further complicated by novel immunotherapy response phenomena, e.g. pseudoprogression, which are being uncovered and are not fully characterized. Current clinical and radiologic assessment strategies are inadequate to interrogate and discern between immunomodulation-influenced response behavior and further diagnostic innovation is warranted to meet the needs of evolving clinical practice in the era of immunotherapy.

## Introduction

Immunotherapy is increasingly establishing itself as the fourth arm of oncologic treatment and its application is actively being translated from the preclinical and clinical trial setting to routine clinical use. Novel and unexpected patterns of treatment response are being uncovered [1,2]. Additionally, radiation therapy may potentially play a significant role in systemic sensitization of immunologic therapy [3] (e.g., the “abscopal response”). A more thorough understanding of the clinical and biological response patterns of such treatments is needed. Such an understanding would potentially be informative toward a “personalized” approach to utilizing immunotherapy and radiation therapy.

Herein, we present and discuss a case of heterogeneous response patterns after combined immunotherapy and comprehensive oligometastasis-directed stereotactic radiation. We consider the possibility of underlying combination immunotherapy and radiation therapy systemic effects in addition to local effects of radiation. Possible paradoxical immunotherapy-related response phenomena are also considered in accounting for the pattern of response observed in this case. Analysis of this case demonstrates the limitations of current clinical and radiologic tools to assess and inform individual patient management given the emerging paradigm of immunomodulation for cancer control.

## Case presentation

**Figure 1:**
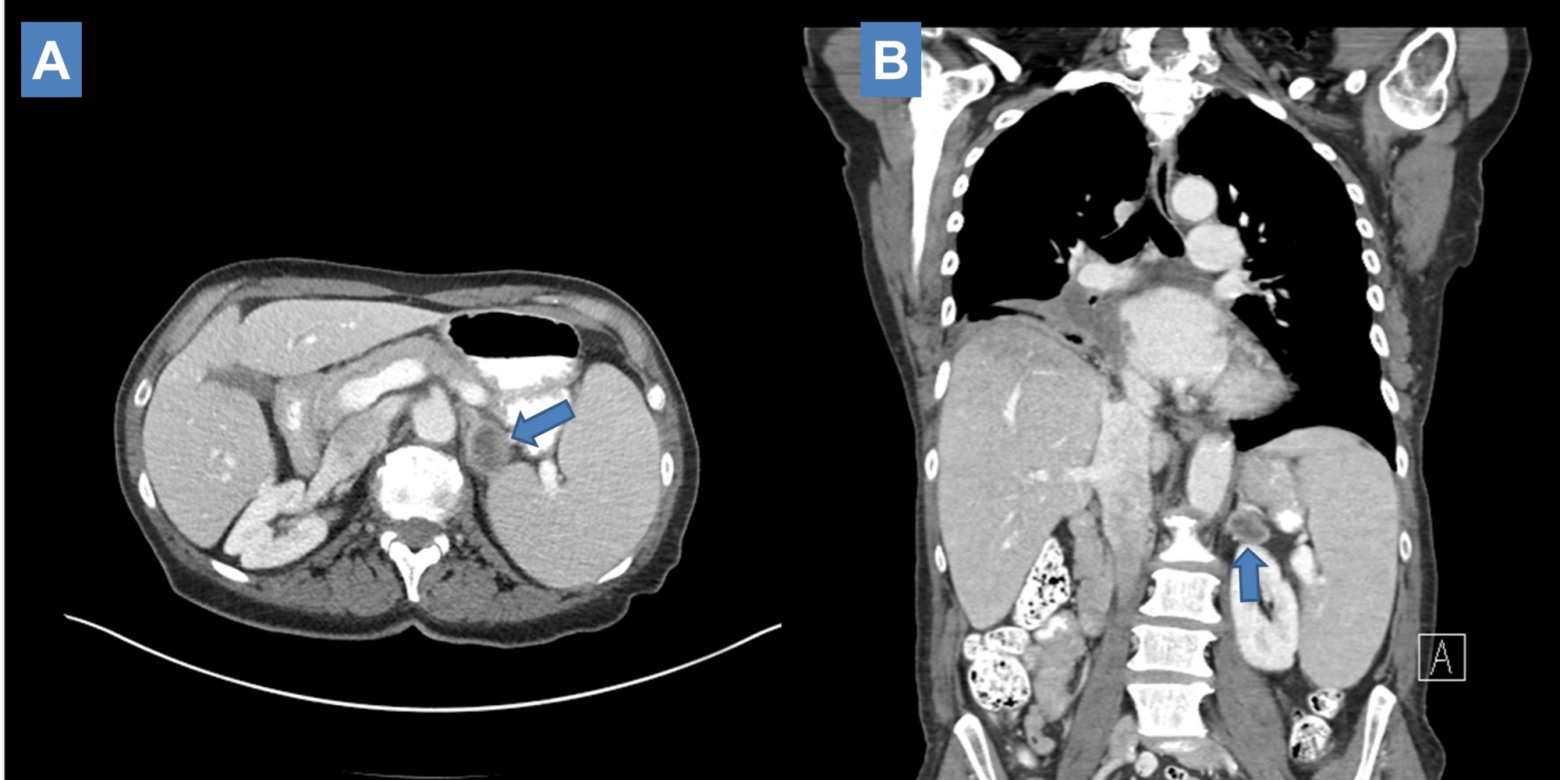
Initial post definitive treatment computed tomography (CT) scan disclosing new left adrenal metastasis (2.8 cm x 2.4 cm). (A: Axial view; B: Coronal view).

Our patient was a 70-year-old female with past medical history significant for non-melanomatous skin cancers and 40 pack-year of smoking who initially presented with complaints of cough and fatigue and was treated with antibiotics for pneumonia. Interval X-ray after non-response to antibiotics showed a right perihilar mass and right middle lobe collapse. Staging computed tomography (CT) of the chest elaborated a 6.5 cm right perihilar mass encasing and obstructing the right middle lobe. Positron emission tomography (PET)/CT and bronchoscopic sampling were undertaken and K-ras positive, PDL-1 unknown adenocarcinoma with involvement of lymph nodes 4R and 7 were confirmed without evidence of distant spread. Imaging had suggested possible invasion of her atrium; however, an echocardiogram did not confirm this finding. Given her Stage IIIB T3 N2 M0 (per AJCC 8th edition) disease, she was offered curative-intent treatment with definitive conventional chemoradiation on a systemic therapy trial randomizing the addition of veliparib, a poly ADP-ribose polymerase (PARP) inhibitor, to conventional carboplatin/paclitaxel during radiation and additionally as part of consolidation. She was treated with 60 Gy using a volumetric modulated arc therapy/intensity modulated radiotherapy (VMAT/IMRT) technique and developed transient esophagitis. Chemotherapy was discontinued during treatment due to poor tolerance; she was taken off the clinical trial. Two weeks after completing radiation treatment, CT imaging demonstrated decrease in size of perihilar primary and mediastinal lymph nodes. However, a left adrenal mass was appreciated measuring 2.8 cm x 2.4 cm (Figure 1).

**Figure 2:**
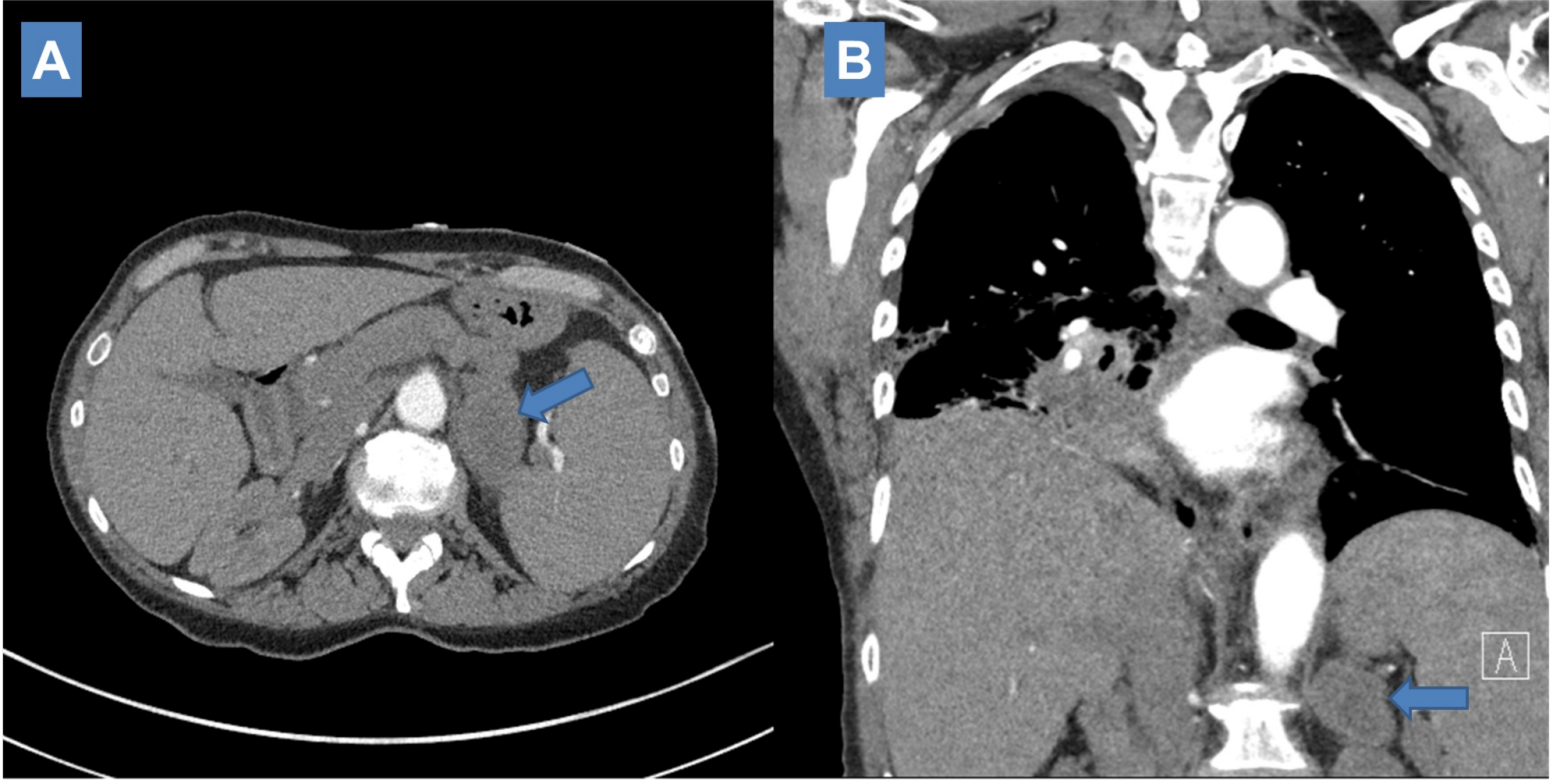
Six-week post definitive treatment computed tomography (CT) scan measuring enlarging left adrenal metastasis (3 cm x 4 cm

She was started on nivolumab and after receiving two doses, approximately six weeks after completing radiation treatment, she was admitted for complaints of shortness of breath, hypoxemia, and acute right-sided rib pain, and was managed with antibiotics for presumed post-obstructive pneumonia on the basis of radiologic findings of increased parenchymal lung patchy opacities and ground glass attenuation distal to the primary on CT. The right perihilar primary appeared stable in size. CT imaging measured the left adrenal mass as appearing larger at 3 cm x 4 cm (Figure 2).

**Figure 3:**
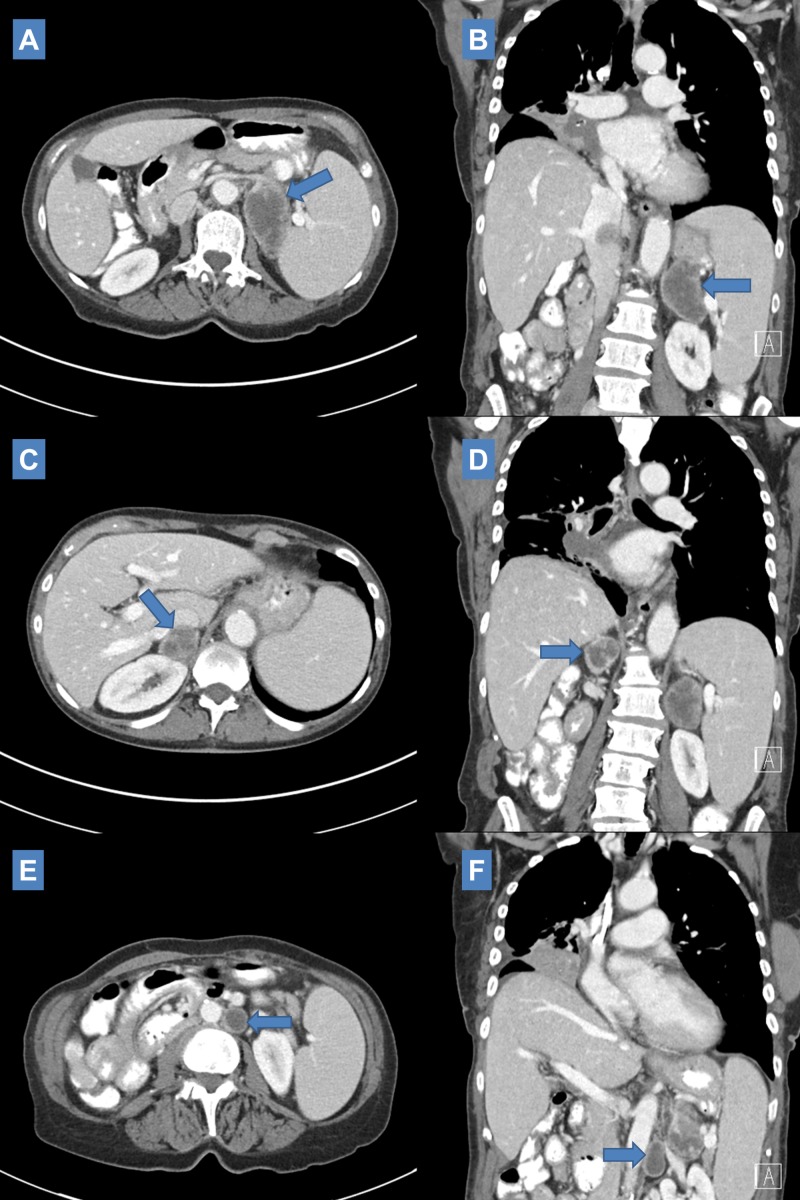
Three-month post definitive treatment computed tomography (CT) scan visualizing enlarging left adrenal metastasis (6.4 cm x 3.7 cm

Interval CT scan three months after completing treatment showed slight decrease in size of the perihilar primary and decrease in size of mediastinal lymph nodes. However, at this point bilateral adrenal involvement was noted (Figure 3A-3D). The previously known left adrenal metastasis appeared enlarged measuring 6.4 cm x 3.7 cm and the newly diagnosed right adrenal metastasis measured 3.2 cm x 2.7 cm. In addition, a newly developed 2.1 cm x 2.1 cm left para-aortic lymph node was disclosed which appeared centrally necrotic (Figure 3E, 3F).

**Figure 4:**
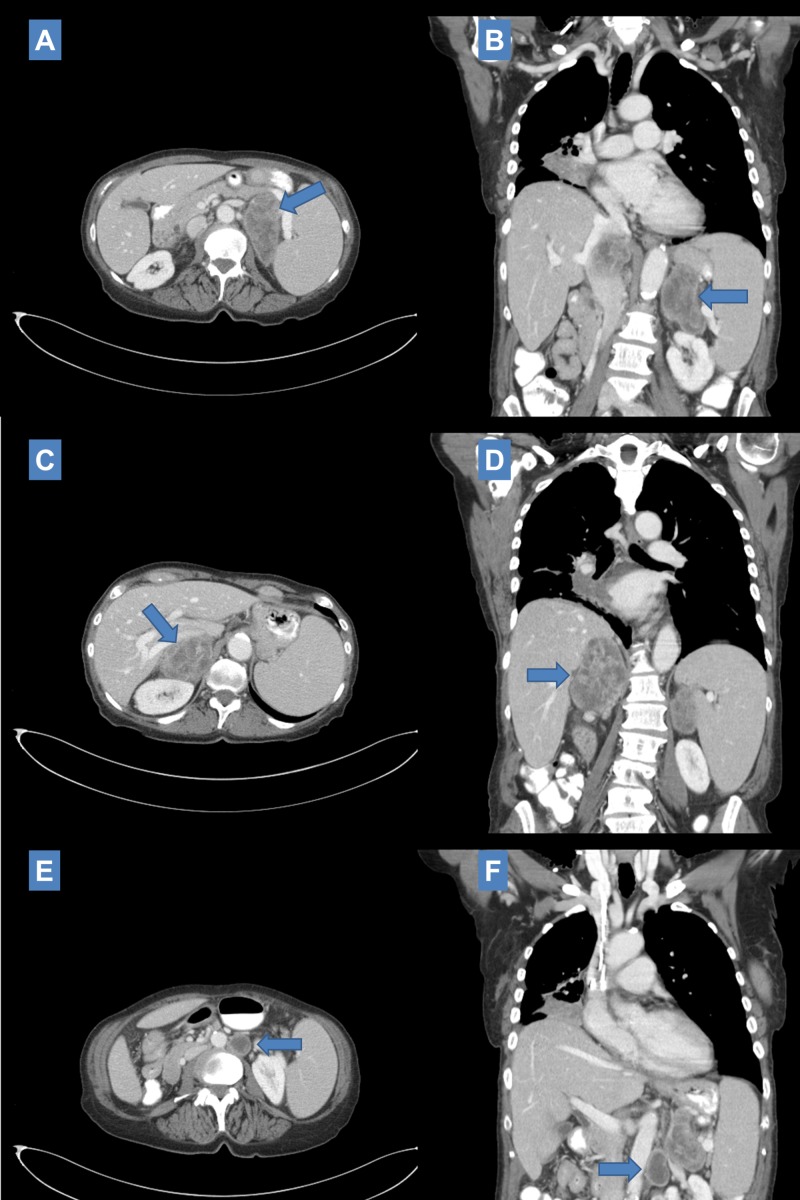
Five-month post definitive treatment computed tomography (CT) scan showing continued enlargement of left adrenal metastasis (8.0 cm x 3.9 cm

Interval CT scan two months later (five months since initial definitive treatment) showed stable size in the lung primary with associated obstructive atelectasis and continued significant increase in size of bilateral adrenal masses (Figure 4A-4D). The left adrenal metastasis measured 8.0 cm x 3.9 cm and appeared heterogeneous; the right adrenal metastasis measured 4.7 cm x 6.5 cm. The left para-aortic lymph node was also noted to have enlarged to 2.5 cm x 2.5 cm (Figure 4E, 4F).

**Figure 5:**
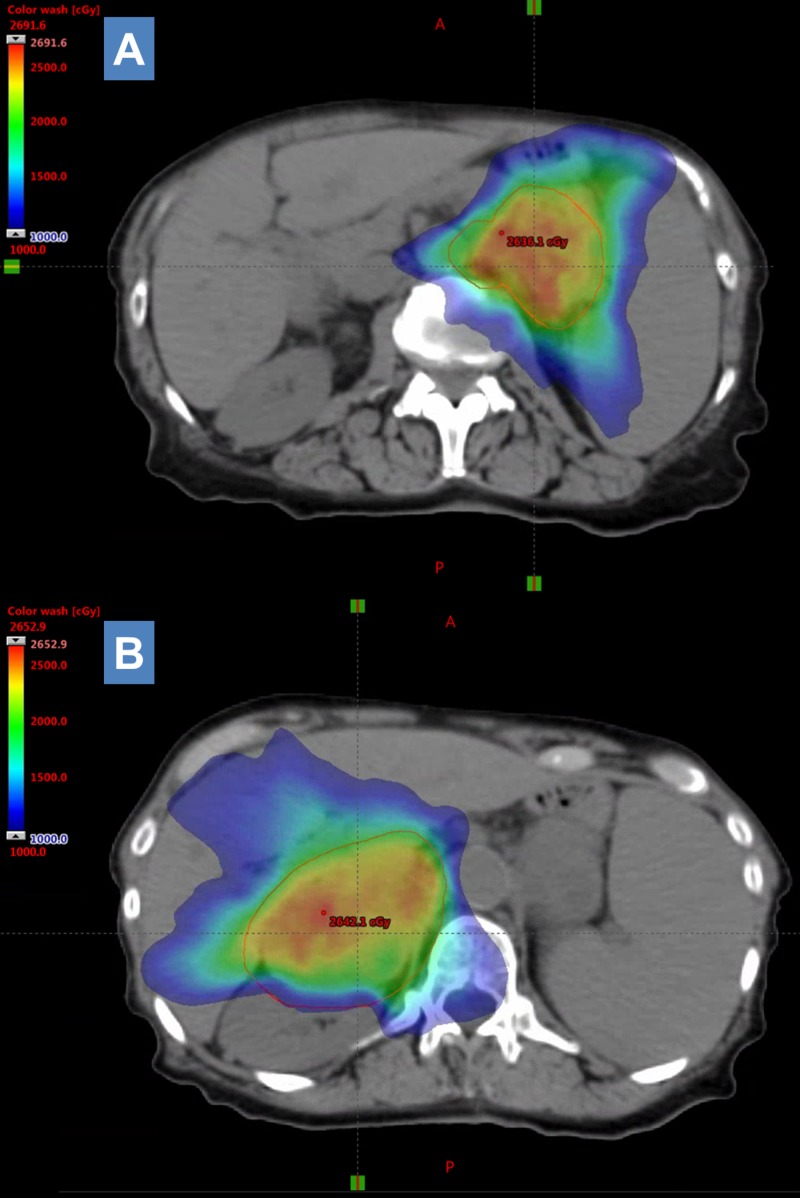
Stereotactic radiation treatment plan selected axial slices representing spatial distribution of dose. Left (A) and right (B) adrenal metastases were treated with stereotactic radiation to a total dose of 25 Gy in five fractions with steep dose drop-off as visualized by the continuous dose gradient overlaying treatment planning computed tomography (CT) scan. The left para-aortic lymph node was included in the left adrenal mass target volume.

**Figure 6:**
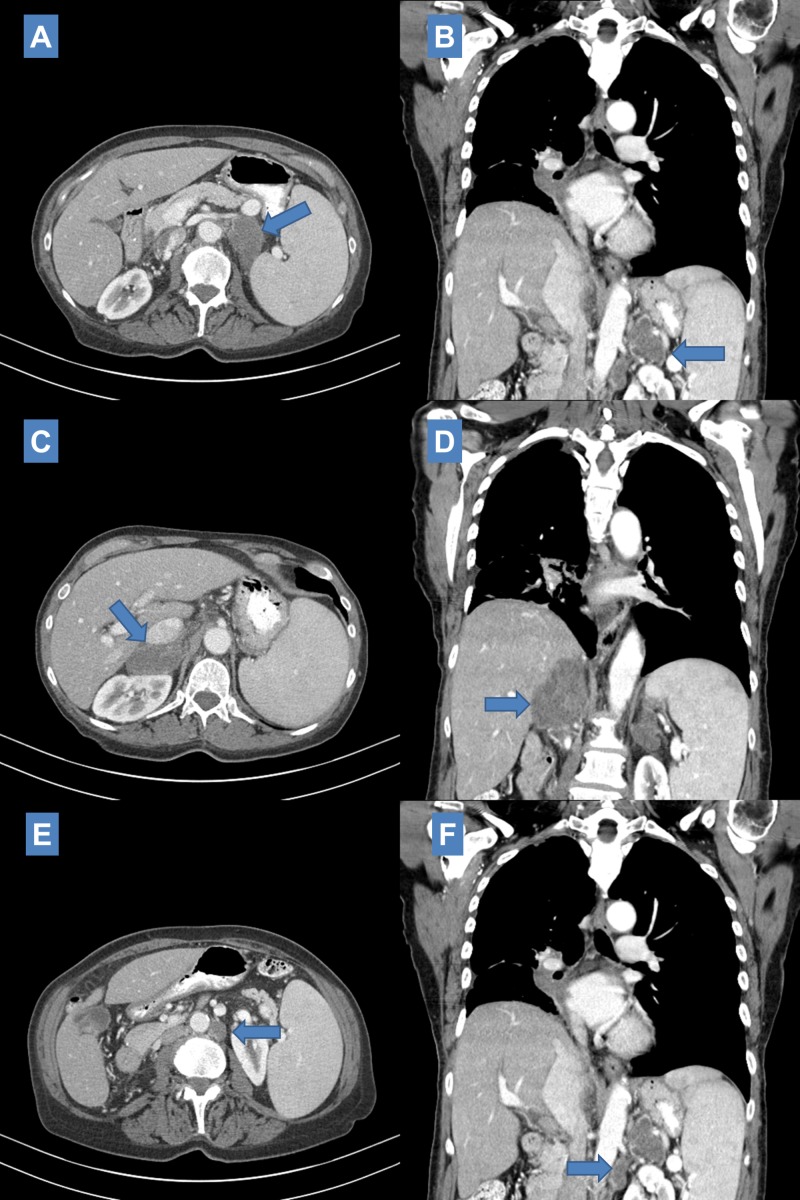
One month post SBRT CT scan showing decrease in size of left adrenal metastasis (4.4 cm x 2.3 cm SBRT: Stereotactic body radiation therapy; CT: Computed tomography.

She subsequently received photon stereotactic body radiation therapy (SBRT) to both adrenal lesions to 25 Gy in five fractions (Figure 5A, 5B) eight months after initial definitive treatment. Retrospective review of the radiation treatment plan shows the left para-aortic lymph node was essentially included in the left adrenal target volume and hence would have received prescription dose. At the time of treatment the left adrenal mass measured 6.9 cm x 3.5 cm, the right adrenal mass measured 6.1 cm x 5.6 cm, and the left para-aortic lymph node measured 2.5 cm x 2.4 cm. CT scan one month after SBRT (nine months after initial definitive treatment) showed the stable lung primary. Decrease in size of both adrenal masses was noted (Figure 6A-6D). The left adrenal mass measured 4.4 cm x 2.3 cm and the right adrenal mass measured 2.0 cm x 6.2 cm. The left para-aortic lymph node decreased in size to 1.4 cm (Figure 6E, 6F).

**Figure 7:**
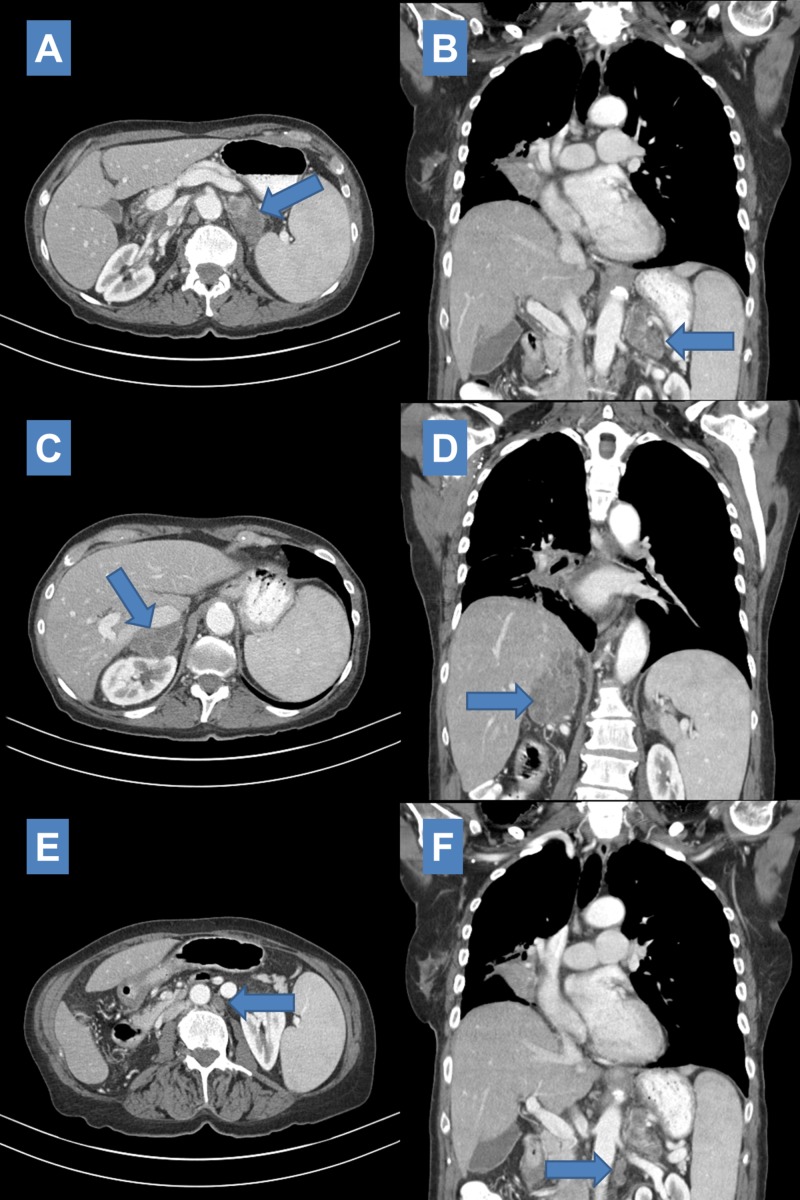
Three-month post salvage SBRT CT imaging scan showing stable size of left (4.4 cm x 2.6 cm SBRT: Stereotactic body radiation therapy; CT: Computed tomography.

Interval CT scan three months after SBRT (11 months after initial definitive treatment) demonstrated stable right hilar mass, stable to decreased size of bilateral adrenal masses (Left: 4.4 cm x 2.6 cm, Right: 4.7 cm x 2.9 cm) and continued decrease in size of left para-aortic lymph node (0.9 cm x 0.8 cm) (Figure 7). There was noted development of irregular nodular opacities at the medial right lung base measuring 12 mm and 10 mm.

**Figure 8:**
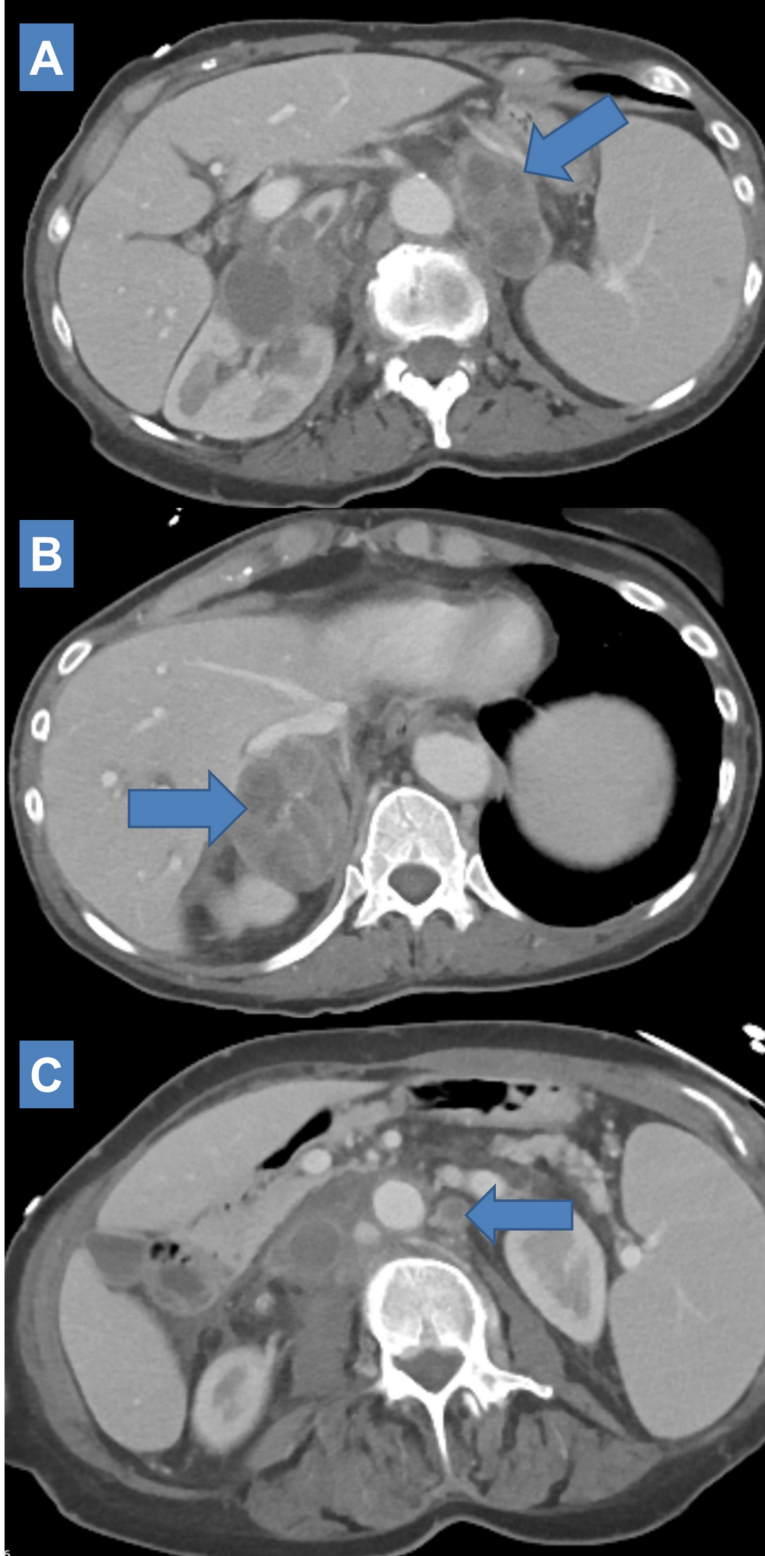
Six-month post salvage SBRT CT imaging scan showing progressive increase in size of left adrenal metastasis to 6.0 cm x 3.0 cm (A: Axial) and right adrenal metastasis to 6.4 cm x 3.5 cm (B: Axial) with the right adrenal metastasis evidencing local invasion (liver, kidney, IVC) and essentially stable size of left para-aortic lymph node (C: Axial). Nivolumab maintenance was held briefly during this time. SBRT: Stereotactic body radiation therapy; CT: Computed tomography; IVC: Inferior vena cava.

Roughly five months after SBRT (13 months since initial definitive treatment) she was admitted with pneumonia which was thought to be post-obstructive. CT imaging without contrast enhancement at this time measured increase in size of bilateral adrenal masses (Left: 4.9 cm x 3.1 cm; Right: 6.2 cm x 3.5 cm). Right perihilar primary was assessed to have enlarged to 2.7 cm x 3.4 cm from 1.9 cm x 2.6 cm with additional note of increase in size of a right lower lobe nodule from 10 mm to 12 mm and increased coalescence of bandline consolidation in the right hilum along the right lower lobe with surrounding interlobular septal thickening. Nivolumab was held. She was discharged and prescribed a long course of antibiotics. She was noted to have slow recovery in performance status with ongoing anorexia and weight loss. Pulmonary stenting was considered and not pursued. An additional dose of nivolumab was given. Follow-up contrast enhanced CT imaging corresponding to six months after SBRT (14 months since initial definitive treatment) demonstrated progressive findings of enlargement of the bilateral adrenal metastases (Left: 6.0 cm x 3.0 cm; Right: 6.4 cm x 3.5 cm) (Figure 8A, 8B) with the right adrenal mass demonstrating new invasion of the right hepatic lobe, upper pole of the right kidney, and direct invasion of the inferior vena cava (IVC). New occlusive thrombus in the IVC from the iliac confluence to the level of the renal veins approximately 12.5 cm in length was noted. Two months after her admission, she was noted to have continued poor performance status, increased work of breathing, poor appetite, and ongoing weight loss in addition to tachycardia and hypotension. She was transferred to home hospice where she eventually passed.

**Figure 9:**
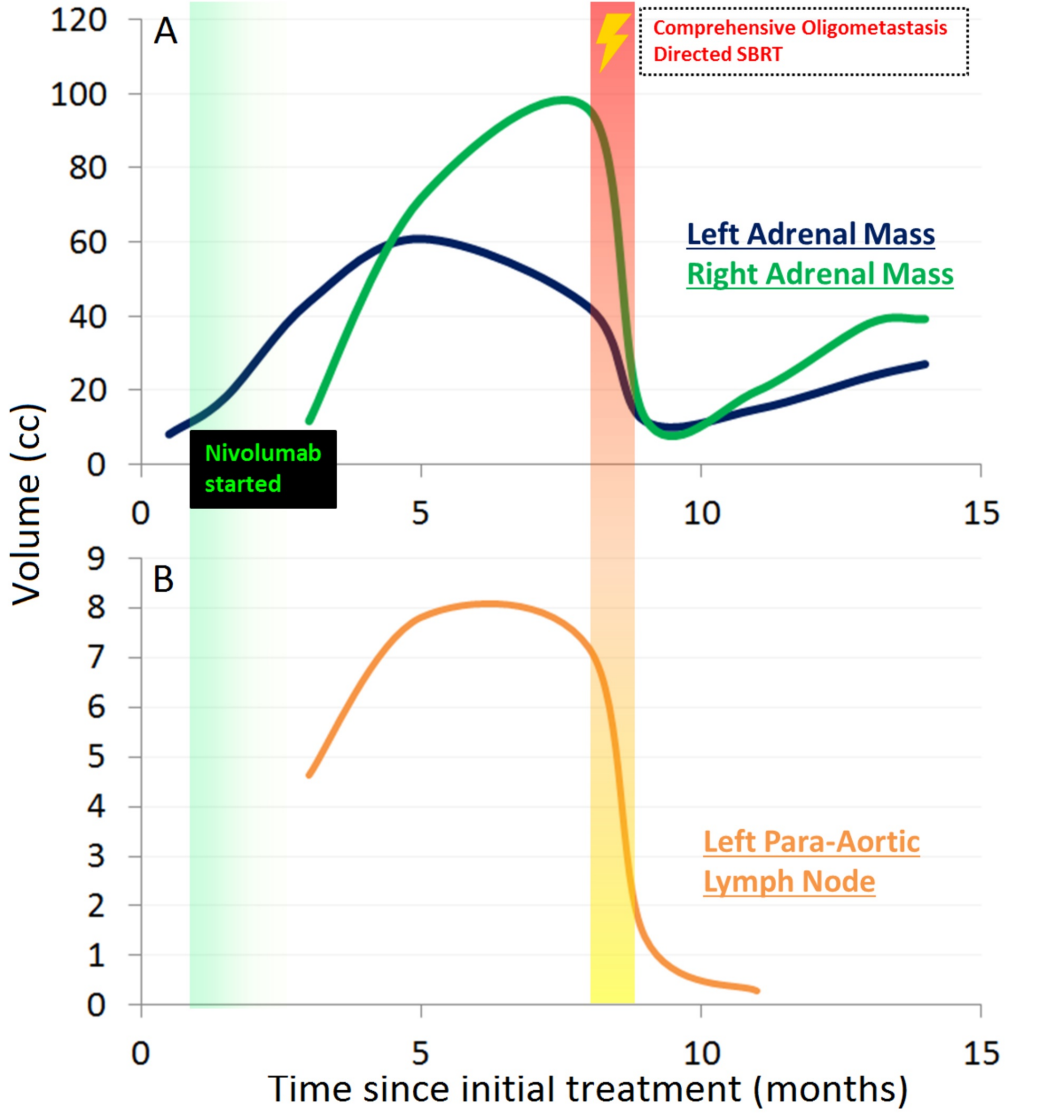
Time course of volumetric changes plotted against time since initial definitive treatment for left and right adrenal masses (A) and left para-aortic lymph node (B) illustrating initial progression and then response across all lesions. Initiation of nivolumab is graphically indicated at approximately one month. Use of salvage SBRT is graphically indicated at eight months. Solid lines plot available empirically measured data abstracted from CT imaging. Volumes are estimated by hemi-ellipsoid calculation: V = Length x Width^2^/2. SBRT: Stereotactic body radiation therapy; CT: Computed tomography.

Figure 9 graphically summarizes the aforedescribed serial time course changes in bilateral adrenal masses (Figure 9A) and left para-aortic lymph node (Figure 9B).

## Discussion

We have reported a case of a patient who presented with PD-L1 unknown initial N2 Stage III non-small cell lung cancer and subsequent synchronous oligometastatic disease whose management with systemic checkpoint inhibitor and consolidative stereotactic radiation demonstrated a dynamic and non-linear time course of treatment effect across multiple radiographically visible lesions. Although this patient ultimately succumbed to local complications at the site of her primary disease, this case is illustrative of the complex nature of individual patient response assessment of immunotherapy, especially as novel response patterns and possible synergistic systemic responses with radiation treatment are considered. This complexity has implications regarding informing clinical decision making and the development of an optimal combinatorial strategy of immunotherapy and radiation treatment. In this discussion, we will analyze the notable observations of the case, hypothesize regarding the possible mechanisms that may account for said findings, and simultaneously discuss the limitations of current clinical assessment strategies and diagnostic tools to assess this scenario.

Truly progressive disease or checkpoint inhibitor-related pseudoprogression?

It is interesting to note that after starting nivolumab for a solitary synchronous metastasis, a paradoxical response was seen in which there was near immediate radiographic progression in the size of her known left adrenal metastasis (Figure 2) followed soon by the emergence of enlarging oligometastatic disease in the contralateral adrenal gland and nearby left-sided para-aortic lymph node (Figure 3). This was followed by apparent plateau in volume increase in two out of three lesions (Figure 9); the right adrenal mass continued to grow, albeit with some slowing, until the delivery of SBRT. This may represent a case of immunotherapy “pseudoprogression.” Pseudoprogression is a phenomenon whereby radiographic increase in size of tumor or tumor burden is noted after initiating checkpoint inhibitor immunotherapy with subsequent reduction in tumor burden [1,2]. Biopsy of these lesions may disclose inflammation or necrosis on pathology. Chiou and Burotto’s summary of the clinical trial experience in observing this phenomenon (mostly from melanoma trials), estimates its occurrence at roughly 10% with checkpoint inhibition and suggests such patterns of immune-related response may have prognostic implication [1].

Grossly, the better than expected survival duration our patient experienced suggests the efficacy of immunotherapy in her case and would support the interpretation that she experienced pseudoprogression. If we were to consider her initial staging as clinical Stage IIIB with subsequent upstaging to Stage IVB (per AJCC 8th edition) we would have expected her median survival to be roughly of seven months [4]; she lived for 15 months. The strength of this interpretation is confounded by the oligometastasis-directed ablative radiation she received. However, several other features of her clinical course would argue that checkpoint inhibition was effective in controlling her disease, at least for some significant amount of time: the lack of early locally progressive disease at her primary site despite suboptimal chemoradiation, the lack of progression to further metastatic sites despite a lengthy interval after initiation of immunotherapy, and her generally maintained performance status until late in her course. On balance, these findings would suggest that she may have experienced pseudoprogression. Biopsies were not obtained after starting immunotherapy in our patient, so ultimately it cannot be pathologically confirmed whether she experienced true progression or pseudoprogression.

Without pathologic confirmation, imaging and clinical algorithms are relied upon to assess treatment response. Recognition of the limitations of Response Evaluation Criteria in Solid Tumors (RECIST) in accounting for immunologic phenomena, such as pseudoprogression, has prompted the development modified response assessment criteria for use with immunotherapy. An older and initially proposed Immune-Related Response Criteria [5] conceptually considered changes in overall tumor burden in determining progression, even if new lesions were to develop. The more recently published iRECIST provide guidelines for modern immunotherapy trials [6] and include more rigorous assessment of new lesions, categorizations of unconfirmed and confirmed progressive disease, sequential time-point assessment of new lesions, confirmation of progressive disease or documentation of why it cannot be confirmed, and documented consideration of overall clinical status. Efforts are underway to validate these assessment criteria.

Application of the aforementioned immune-related response criteria would have likely still categorized our patient as having “true progression.” Again, without pathologic confirmation one cannot be sure. It may be that the crude volumetric and radiographic methods, on which these response criteria approaches are based, are unlikely to capture the vigorous and dynamic physiology underlying the efficacy and clinical patterns of these immune responses. In their review of novel treatment response phenomena after checkpoint inhibition, Wang et al. describe the wide published range of observed increases in tumor burden during pseudoprogression (up to 163% noted thus far), the wide timeframe over which the pseudoprogression may evolve (beyond six months noted in some cases), and hyperprogression (i.e., acceleration of tumor proliferation) which is another highly dynamic paradoxical response [2]. Hyperprogression may be associated with age-related changes in T-cell immunity, changes in oncogenic signaling (e.g., MDM2 and EGFR), and previous radiation in a patient receiving a checkpoint inhibitor. Notably, almost all instances of hyperprogression have been noted to be locoregionally concordant to a previously irradiated field and it has been hypothesized radiation-related tumor microenvironment changes can prime an “immune escape” response to immunotherapy. In a further highlight of the varied presentation of pseudoprogression, a recently published case report and discussion by Vrankar and Unk described a case of a 67-year-old female presenting with extensively metastatic non-small cell lung cancer treated with palliative radiation and chemotherapy with subsequent progression to new metastatic sites; biopsy of a metastatic site disclosed 100% PD-L1 enrichment and a single cycle of pembrolizumab was administered [7]. The patient quickly clinically deteriorated to performance status 4 and was transferred to palliative care. At great surprise to the authors, three months later the patient was back to performance status 1 and subsequently continued on immunotherapy and found to have almost complete response. This is an illustrative example of what appears to be dramatic recovery after severely symptomatic pseudoprogression due to initiation of checkpoint inhibition immunotherapy of extensively metastatic disease. A similar arc of worsening and improvement driven by immunotherapy response may have been similarly observed in the three tracked limited metastatic lesions in our patient. Suffice to say, currently utilized clinical tools, both algorithms/nomograms and conventional imaging modalities, are far from adequate to interrogate these immune processes.

Did radiotherapy elicit a systemic response?

Though two of the three lesions appeared to plateau in their growth by eight months after initiating immunotherapy, it was determined at this point to use SBRT for comprehensive salvage. After SBRT was administered, the tracked lesions continued to demonstrate a mixed pattern of response. It is interesting to consider to what extent local and synergistic systemic effects were at play and whether this could be precisely assessed. On the basis of emerging clinical and preclinical evidence [3,8,9], it is reasonable to presume that an abscopal systemic response was effected to a certain degree in our patient. Interest in the abscopal effect of radiation has experienced a resurgence in recent years since the publication of a seminal case report of distant disease response after localized radiation in a melanoma patient on CTLA-4 blockade which correlated temporally with changes in humoral immunologic factors [10]. Intensive preclinical investigation into the molecular cross-talk between radiobiology and immunology [3,8] and clinical investigation into combining radiation and immunotherapy are underway [9] toward the goal of optimizing the clinical deployment of radiation (i.e., targets, dose/fractionation, sequencing) [11] and fully exploiting its immunologic systemic effects. It has been suggested that hypofractionated radiation, such as which our patient received, may be optimal toward eliciting such systemic responses. Obvious response in untreated sites has been focused upon as surrogate evidence for an abscopal effect in an individual patient; however, given all sites were treated in our patient this approach to assessing systemic response is not possible.

Comprehensive oligometastasis-directed ablative radiation, which has been increasingly validated for clinically significant endpoints in prospective study [12,13], may have had an independent contribution toward the outcome of our patient and is a notable aspect of this case. A prognostic nomogram for oligometastatic non-small cell lung cancer patients, generated on the basis of retrospective individual patient data meta-analysis of 757 patients treated with comprehensive stereotactic ablative radiation [14], would categorize our patient into the highest risk subgroup (synchronous and node positive disease). Interestingly, our patient’s outcome roughly matches the nomogram’s estimation. Though much focus has been the potential of combining immunotherapy with ablative radiation to a single site or to only a few sites of metastatic disease, comprehensive oligometastasis-directed stereotactic radiation may be the optimal strategy by both empiric clinical and mechanistic immune/molecular rationales. Indeed, there has been argument made for moving away from inducing abscopal effects by single-site radiation and instead to utilize comprehensive radiation of all visible lesions to increase the probability of effective immune priming [15]. Immune checkpoint inhibition and comprehensive multi-site stereotactic radiation may represent a convergent strategy. This would appear to be conceptually congruent with recently published research examining clinical metastasis specimens, in this case colorectal liver metastases, suggesting integrative molecular subtyping may define a curable oligometastatic state apparently driven by an immune-enriched profile [16].

Response heterogeneity across metastatic sites and time

Our patient received SBRT to all her obvious sites of disease which would have theoretically been thought to provide her both maximal cytoreductive benefit and maximum systemic immune potentiation. However, it does not appear her post-SBRT course evolved along those expectations. One can speculate as to what molecular/tumor-immune processes transpired in the latter part of her course and if any clues can be gleaned from the time course of her imaging across metastatic sites. Ultimately, she passed of apparent ongoing post-obstructive symptoms with significant signs of systemic illness. This may have been due to local progression at her primary site as last available chest CT had suggested growth in her right perihilar lesion which had otherwise been radiographically stable for over a year after receiving suboptimal chemoradiation. Interestingly, this was preceded by apparent radiographic growth in her bilateral adrenal masses which had initially demonstrated significant decrease in size after SBRT (Figure 6). At the time of last follow-up, these continued to appear radiographically progressive from nadir: the right adrenal metastasis developed on apparent invasive features (Figure 8B) while the left adrenal metastasis demonstrated less rapid regrowth and no evidence of local invasion (Figures 8A, 9A). This was observed concomitant to apparent sustained response in the left para-aortic lymph node (Figures 6E, 6F, 7E, 7F, 9B). Can the mechanisms underlying this heterogeneity in short-to-medium response to ablative radiation be understood and do systemic interactions with immunotherapy play role? A number of mechanisms may be hypothesized. One would be the patient had experienced sustained systemic immune response at the primary site after initial definitive treatment and subsequent initiation of checkpoint inhibition which was eventually overcome after several months. The time-course of response seen in the adrenal metastases particularly suggests the possibility of an on-going dynamic immunologic response. An atypical pattern of immediate volumetric treatment response followed quickly by rebound growth was seen. This runs counter to several published experiences demonstrating excellent durable local control rates for adrenal metastasis treated with SBRT; if local progression were to occur, it would typically occur after several months, not immediately [17]. One can speculate whether an immune escape/hyperprogression phenomenon, as previously discussed, was observed and whether it was possibly potentiated by radiation treatment.

Ultimately, it may be that the patient’s intrinsically aggressive cancer biology continued to evolve through the course of her treatment, recalling there was suspicion of atrial invasion in the beginning which would have made her T4 from the outset. However, this consideration is in contrast to her better than expected outcome on a stage for stage basis and lack of development of further metastatic sites. The most reasonable conclusion may be that there is a spectrum immune control activity (i.e., not binary) and tumor proliferative behavior/aggressiveness which may be spatially heterogeneous, dynamically evolve over time, and impacted by both immunotherapy and radiation in varied and possibly non-intuitive ways. Suffice to say, it is difficult to sort out the exact nature of her tumor burden and immune physiologic status with the available conventional clinical data.

Future directions for immunotherapy personalization

On the basis of aforedescribed analysis of this illustrative case, it is our suggestion that immune treatment response be conceptualized as an on-going dynamic and multi-dimensional process that may be amenable to proper understanding of clinical utility in individual patients in a quantitative and functional way via advanced imaging techniques and the integration of newer adjunctive diagnostic technologies. Our patient’s case highlights a number of junctures where more elucidating information than is clinically available would likely be impactful, particularly with regard to delivering radiation. Rapid translation, integration, and clinically tuning of diagnostic technologies are poised to make immediate and significant clinical impact. Integrative approaches combining novel immuno-PET-based molecular imaging techniques [18], humoral immunophenotyping [19], and circulating tumor DNA assessment [20] may provide the foundation for biomarker-driven approaches toward immunomodulation and may have stronger implications for guiding locally ablative radiation treatment in combination with immunotherapy.

## Conclusions

We discussed an illustrative case of the difficulty of immunotherapy treatment response assessment, which was made more difficult by confounding and likely synergistic effects of concomitantly delivered ablative radiation treatment. Current clinical and radiologic assessment strategies are inadequate to assess immunotherapy treatment response and further innovation and translation of diagnostic approaches and techniques are warranted to meet the needs of evolving clinical practice in the era of immunotherapy.
